# Mantle Cell Lymphoma in the Thyroid: A Rare Presentation

**DOI:** 10.1155/2017/6749801

**Published:** 2017-12-25

**Authors:** Uzma Mohammad Siddiqui, Sarika N. Rao, Pallavi Kanwar Galera, Nahida Islam, Mira S. Torres

**Affiliations:** ^1^Division of Endocrinology, University of Massachusetts Medical School, 55 Lake Avenue N., Worcester, MA 01655, USA; ^2^Section of Endocrinology, MedStar Washington Hospital Center, 110 Irving St NW, Suite 2A-72, Washington, DC 20010, USA; ^3^Department of Pathology, University of Massachusetts Medical School, Biotech 3, 1 Innovation Drive, Worcester, MA, USA; ^4^Division of Hematology and Oncology, University of Massachusetts Medical School, 55 Lake Avenue N., Worcester, MA 01655, USA

## Abstract

**Background:**

While 2% of all extranodal Non-Hodgkin Lymphomas present in the thyroid, there exists insufficient data to describe the incidence of mantle cell lymphoma in the thyroid. A case series of 1400 patients revealed that <1% of thyroid lymphomas may be MCL; hence better understanding of the disease course is essential.

**Patient Findings:**

A 65-year-old female was referred for a multinodular goiter. Multiple fine needle aspirations from the dominant right nodule were consistent with Hashimoto's thyroiditis and flow cytometry was negative. Due to progressing dysphagia, she underwent total thyroidectomy.

**Summary:**

Pathology revealed MCL with mantle zone growth pattern in the right thyroid. Flow cytometry showed monoclonal B cells comprising 9% of total cells. The Ki-67 index was 10%. She was diagnosed as having stage IIE MCL and offered conservative management by medical oncology, given that she had no B symptoms.

**Conclusion:**

Though chemotherapy is the treatment of choice in MCL, a subset of patients with low-grade disease may be observed. As in our patient, mantle zone growth pattern and a Ki-67 index < 10% suggest a favorable prognosis. A diagnosis of primary MCL in the thyroid remains rare and staging modalities as well as treatment options continue to evolve.

## 1. Introduction

Primary thyroid lymphomas (PTL) are an infrequently encountered diagnosis. Studies have suggested that less than 5% of all thyroid malignancies may be PTL [1]. Mantle cell lymphoma (MCL) is one such type: it is an aggressive variant of the Non-Hodgkin Lymphomas (NHL), accounting for less than 10% of all NHL [2]. While most MCL present in lymph nodes and may have extranodal involvement, less than a quarter of all cases present primarily in extranodal sites [3]. These extranodal sites include gastrointestinal tract, liver, spleen, peripheral blood, and central nervous system [2, 4]. A population-based study of PTL in about 1400 patients in the United States revealed that only about 1% of patients had MCL [5].

Mantle cell lymphomas primarily originate from the pregerminal B cells of the mantle zone. They have been consistently shown to have cyclin D1 overexpression, the majority of which are caused by a bcl rearrangement t(11;14) [6, 7]. This overexpression leads to unregulated cellular proliferation and growth. Treatment with immunochemotherapy is offered for most patients, while radiation therapy and stem cell transplantation are used in selected patients [8].

Although oncologists describe MCL as more aggressive than most NHL, the course of our patient was actually quite favorable. This, accompanied by the lack of detailed clinical descriptions of MCL presenting primarily in the thyroid in the existing literature, makes this case valuable for both educational and future research purposes.

## 2. Case Presentation

A 58-year-old female, with a past medical history of Hashimoto's thyroiditis, was referred to the Endocrinology Clinic for evaluation of a multinodular goiter. This goiter was initially discovered in 2000. She had previously undergone fine needle aspiration (FNA) of a dominant thyroid nodule (2.4 cm) on the right lobe. The pathology for this had returned benign, consistent with Hashimoto's thyroiditis, and had a negative flow cytometry. From 2000 to 2008, multiple FNAs from the same nodule were consistent with the same result.

**Figure 1:**
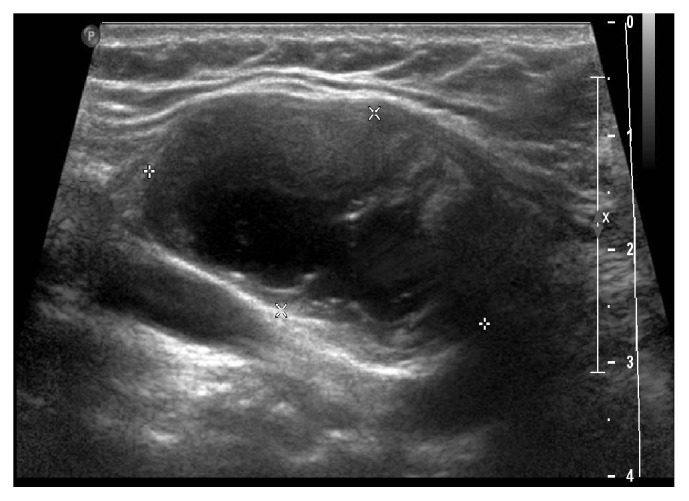
Right-sided dominant thyroid nodule seen on thyroid ultrasonography.

On presentation to our clinic in 2008, she was essentially asymptomatic. She was clinically euthyroid. She did not have any dyspnea, dysphagia, or dysphonia. On exam, she had right-sided fullness of her thyroid and had an overall nodular gland. Medical history other than Hashimoto's thyroiditis and the multinodular goiter included Type 2 diabetes mellitus, osteopenia, hypertension, and hyperlipidemia. She was a former smoker and had quit at the age of 34. Of note, her mother had thyroid cancer, but the details of this were not available. Her medications at that time included levothyroxine, metformin, atorvastatin, lisinopril, and vitamin D. Lab work revealed thyroid stimulating hormone was 1.50 uUI/mL (0.28–3.89) with a free thyroxine level of 0.98 ng/dl (0.58–1.64). Her right nodule remained stable until November 2014, when it was noted to have grown to 3.8 cm (Figure 1). At that time, she complained of dysphagia and neck discomfort. Given these symptoms, she underwent total thyroidectomy in February 2015.

**Figure 2:**
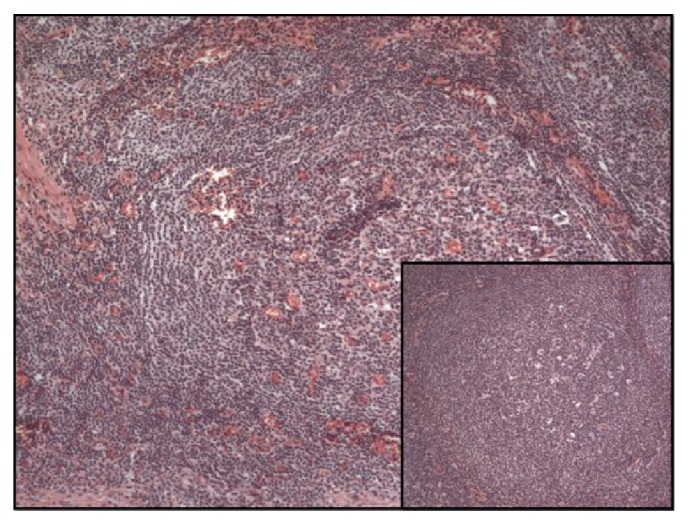
Mantle cell lymphoma involving the thyroid and prelaryngeal lymph node (insert), stained with Hematoxylin and Eosin (100x).

**Figure 3:**
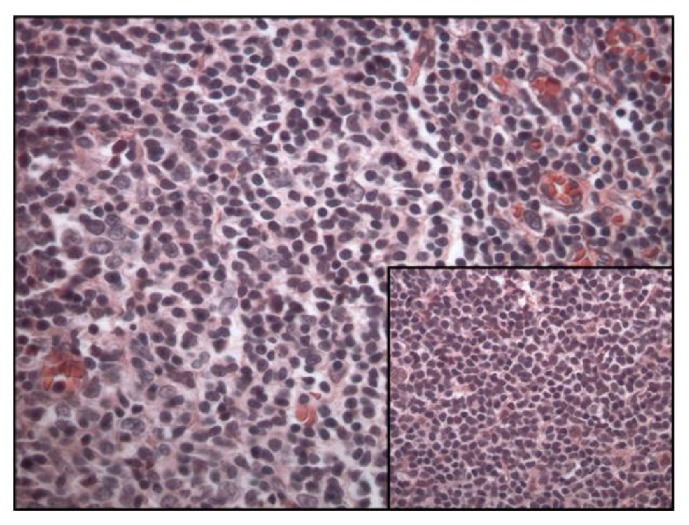
Mantle cell lymphoma involving the thyroid and prelaryngeal lymph node (insert), stained with Hematoxylin and Eosin (400x).

On gross examination, a large cystic nodule (4.2 cm) was identified in the right lobe of the thyroid with multiple smaller nodules in the left lobe. Sections from the right thyroid nodule revealed dense lymphocytic infiltrate, which formed variably sized follicles with germinal centers and marked expansion of mantle zones surrounding the germinal centers. The neoplastic cells in the mantle cell zones were small with irregular nuclear contours, condensed chromatin, and scant cytoplasm. A Delphian lymph node excision done at the same time revealed similar involvement by mantle cell lymphoma (Figures 2 and 3).

**Figure 4:**
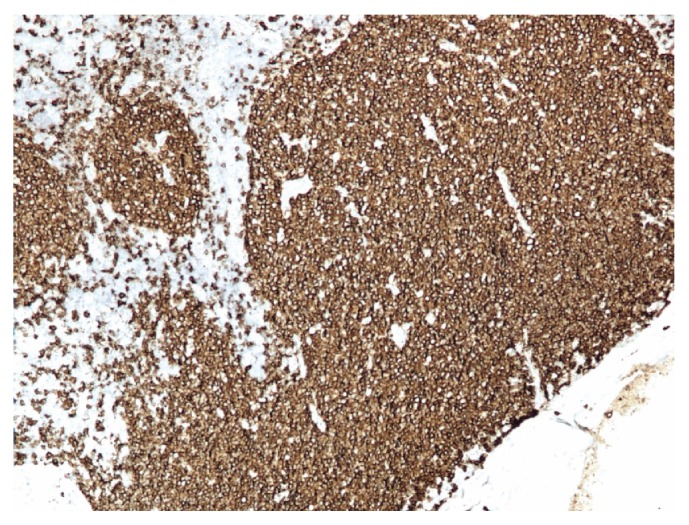
Mantle cell lymphoma (100x). Positive staining with CD20.

**Figure 5:**
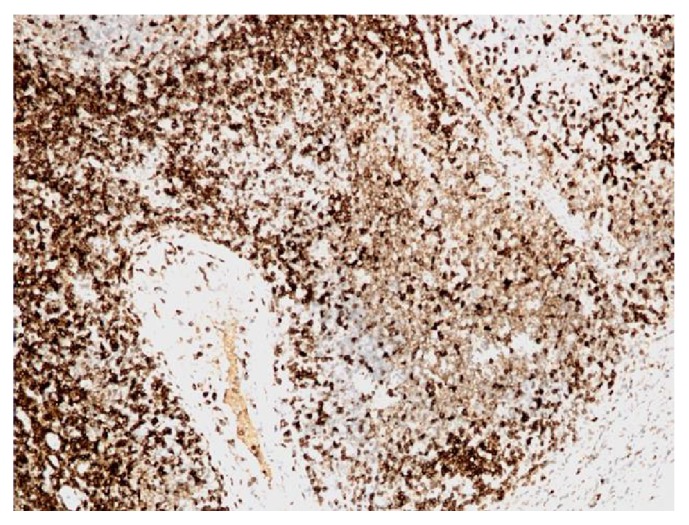
Mantle cell lymphoma (100x). Positive staining with CD5.

**Figure 6:**
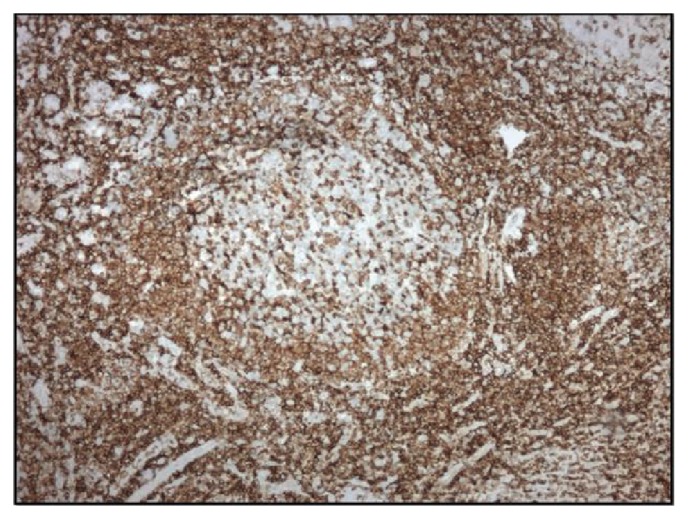
Mantle cell lymphoma involving thyroid (100x). Positive staining with CD43.

**Figure 7:**
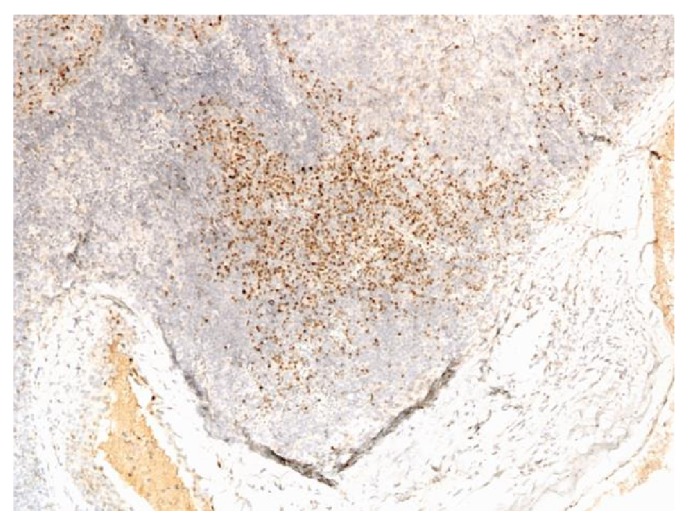
Mantle cell lymphoma (100x). Positive staining with cyclin D1.

**Figure 8:**
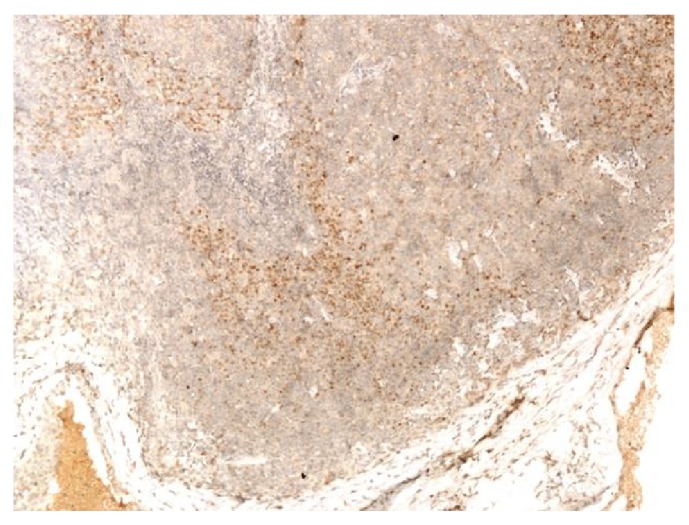
Mantle cell lymphoma (100x). Positive staining with SOX 11.

**Figure 9:**
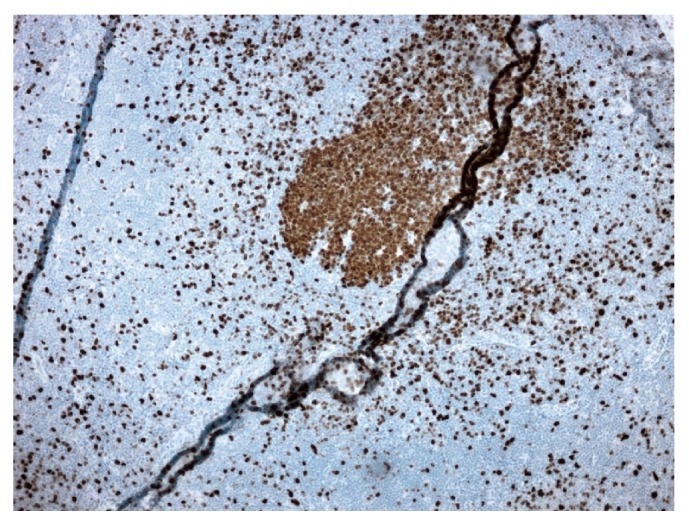
Mantle cell lymphoma (100x). Ki67 staining.

Immunoperoxidase stains revealed that the expanded mantle zone cells (neoplastic B cells) were positive for CD20 (Figure 4), CD5 (Figure 5), CD43 (Figure 6), cyclin D1 (Figure 7), and SOX11 (Figure 8). Cyclin D1 and SOX11 also highlighted scattered interfollicular neoplastic B cells. Concurrent flow cytometric analysis identified CD19+, CD20 bright+, CD5+, and CD10− monoclonal B cells with lambda light chain restriction. These findings were consistent with mantle cell lymphoma involving thyroid with predominantly mantle zone growth pattern. FISH (fluorescence in situ hybridization) analysis done on sections of the lymph node showed a CCND1-IGH rearrangement, which occurs due to chromosomal translocation t(11;14). The Ki-67 index was 10% (Figure 9). Sections of the left lobe were negative for cyclin D1 and SOX11 stains.

She was referred to medical oncology and underwent a bone marrow aspiration for staging. Her calculated MIPI (MCL International Prognostic Index) was 3 and her International Prognostic Index (IPI) was 1. This made her disease of low risk.

She was diagnosed as having stage IIE MCL as she only had disease in the thyroid and prelaryngeal lymph node and the bone marrow biopsy was negative. Therapy was not recommended because the patient was asymptomatic. Under close surveillance, there has been no evidence of tumor recurrence or progression.

## 3. Discussion

PTL account for a small number of all thyroid malignancies, between 2 and 5% of all cases [1, 11]. These are usually Non-Hodgkin Lymphomas, and Hodgkin's Lymphoma in the thyroid is exceedingly rare. PTL have been shown to have preponderance in the elderly female population, presenting mostly after the 5th decade of life [12]. It usually presents as a rapidly enlarging painless mass, often with compressive symptoms such as stridor, shortness of breath, and dysphagia [11, 12]. Hashimoto's thyroiditis is often implicated as a risk factor for the development of PTL, where studies have suggested that the risk is increased almost 60-fold compared to the general population [13].

While the most common form of PTL is a diffuse large B cell lymphoma, MCL is a rare variant. MCLs are usually diagnosed at an advanced stage, with mostly extranodal involvement [2]. The diagnosis is established by cytology and distinguished via immunophenotyping. The detection of t(11;14) by either karyotyping or FISH is very useful in making this diagnosis. Another Non-Hodgkin Lymphoma that is often difficult to differentiate from MCL is the marginal zone lymphoma (MZL). The MZL is differentiated into nodal, extranodal, and splenic types [14]. The MZL derives its name from the origin of the lymphoma in the marginal compartment of lymphoid tissues, while, similar to MCL, these are positive for B cell markers CD-20 and CD-22; in contrast the MZLs are negative to CD5. Additionally, the MZLs do not carry the hallmark chromosomal translocation of t(11;14) that is seen in MCL [7, 15].

MCLs are classified as an aggressive lymphoma, with median survival of 3–5 years [16]. Prognosis is difficult to quantify and several prognostic indices exist. One of the previously used prognostic indices is the International Prognostic Index (IPI) [17]. This index accounts for age of patient, stage of disease, serum lactate dehydrogenase, performance status, and presence of extranodal sites. More recently, the MIPI is being used to help risk-stratify patients with MCL at diagnosis. This index utilizes age, performance status, lactate dehydrogenase, and white cell count [18].

Treatment options for MCL have been evolving. They have traditionally been based on chemotherapy with CHOP regimens (cyclophosphamide, vincristine, doxorubicin, and prednisone). More recently patients have been also treated with immunotherapy (rituximab) and autologous stem cell transplantation. Other agents being used are bendamustine and lenalidomide plus rituximab [19]. Relapsed MCL is considered to be even more challenging to treat and other options are available under clinical trials, including ibrutinib, bortezomib, and lenalidomide. Radiotherapy has also been effectively used in the treatment of localized or refractory MCL [20]. However, not all patients require treatment up front as up to a third of MCL patients may be selected for deferred treatment based on low IPI indices [21]. While the MIPI can be variable, when combined with the Ki-67 index, it can provide a more reliable tool for risk assessment, which is called the MIPI-c index. The Ki67 antigen is a marker of cellular proliferation and therefore useful in prognostications. One study revealed that the best survival rate was with Ki-67 index of less than 10% [16]. Apart from the MIPI index and the Ki-67 index, a third marker of indolent disease is the absence of SOX11. This is a nuclear transcription factor and, if present, is implicated in tumorogenesis that is seen in aggressive MCL. However, when absent, it may correlate with improved survival. MCL that is SOX-11 negative may be mostly nonnodal presentation. The absence of SOX11 expression not only has been linked to indolent disease, but also is with improved survival. Other indicators of good prognosis mentioned in the literature are mutated IGHV gene and lack of mutations in 17p/TP 53 [22].

The presence of MCL primarily located in the thyroid is scarcely reported. It is unclear whether the above-mentioned prognostic indices and treatment regimens apply to MCL that presents as a PTL. One case report highlighted the discovery of MCL in the thyroid after a primary presentation in the gastrointestinal tract [9]. This patient had a low Ki-67 index [10–15%] and had good survival with continued surveillance. Another case report demonstrating the presentation of MCL as a PTL did not have any other sites of disease; however it did have a high Ki-67 index [>30%]. The patient required 8 courses of immunochemotherapy (rituximab-cyclophosphamide, epirubicin, vincristine, and prednisone) and achieved complete remission [10] (see Table 1).

The description and clinical course of our patient appear to be those of an indolent MCL, but whether she will require treatment in the future is a question that remains unanswered. In the absence of extensive data on the clinical outcomes of MCL presenting as a PTL, more cases are needed to better understand the disease course and treatment options, as well as overall prognosis. It is also important to note that though chemotherapy is the treatment of choice for MCL, a small subset of patients with low-grade disease may be observed. As in our patient, mantle zone growth pattern and a Ki-67 index < 10% suggest a favorable prognosis. Of note, the two previously reported cases of MCL in the thyroid also exhibited no aggressive disease. A diagnosis of primary MCL in the thyroid remains rare and staging modalities as well as treatment options continue to evolve.

## Figures and Tables

**Table 1:** Comparing our patient to another 2 cases reported in literature.

	Our patient	Hojilla et al. [9] (2016)	Guastafierro et al. [10] (2010)
Age of patient at diagnosis	63	59	59

Presence of Hashimoto's thyroiditis	Present	Presence of hypothyroidism	Present

Stage of disease	Stage II EA	?	Stage I EA

Location of distant metastases	None	Involvement of colon	?

Ki-67 index	10% (lymph node)	10–15% (in the colon)	>30%

Treatment	Surveillance	Surveillance	8 courses of R-CEOP (rituximab-cyclophosphamide, epirubicin, vincristine, and prednisone), followed by rituximab for 2 years

Survival until publication	Progression-free for 2 years	Progression-free for 2 years	Relapse-free for 4 years

## References

[1] Sharma A., Jasim S., Reading C. C. (2016). Clinical presentation and diagnostic challenges of thyroid lymphoma: a cohort study. *Thyroid*.

[2] Goy A. (2016). Mantle cell lymphoma: is it time for a new treatment paradigm?. *Hematology/Oncology Clinics of North America*.

[3] Argatoff L. H., Connors J. M., Klasa R. J., Horsman D. E., Gascoyne R. D. (1997). Mantle cell lymphoma: a clinicopathologic study of 80 cases. *Blood*.

[4] Romaguera J. E., Medeiros L. J., Hagemeister F. B. (2003). Frequency of gastrointestinal involvement and its clinical significance in mantle cell lymphoma. *Cancer*.

[5] Graff-Baker A., Roman S. A., Thomas D. C., Udelsman R., Sosa J. A. (2009). Prognosis of primary thyroid lymphoma: demographic, clinical, and pathologic predictors of survival in 1,408 cases. *Surgery*.

[6] Swerdlow S. H., Yang W.-I., Zukerberg L. R., Harris N. L., Arnold A., Williams M. E. (1995). Expression of cyclin D1 protein in centrocytic/mantle cell lymphomas with and without rearrangement of the BCL1/Cyclin D1 gene. *Human Pathology*.

[7] Rimokh R., Berger F., Delsol G. (1994). Detection of the chromosomal translocation t(11;14) by polymerase chain reaction in mantle cell lymphomas. *Blood*.

[8] Abrahamsson A., Albertsson-Lindblad A., Brown P. N. (2014). Real world data on primary treatment for mantle cell lymphoma: A Nordic Lymphoma Group observational study. *Blood*.

[9] Hojilla C. V., Rajab A., Craddock K. J., Rotstein L. E., da Cunha Santos G. (2016). Mantle cell lymphoma involving the thyroid: a case report. *Cytopathology*.

[10] Guastafierro S., Falcone U., Celentano M. (2010). Primary mantle cell lymphoma of the thyroid. *Leukemia Research*.

[11] Logue J. P., Hale R. J., Stewart A. L., Duthie M. B., Banerjee S. S. (1992). Primary malignant lymphoma of the thyroid: A clinicopathological analysis. *International Journal of Radiation Oncology, Biology, Physics*.

[12] Ruggiero F. P., Frauenhoffer E., Stack B. C. (2005). Thyroid lymphoma: a single institution’s experience. *Otolaryngology—Head and Neck Surgery*.

[13] Kishanprasad H., Hegde P., Chandrika R., Jayaprakash K. (2014). Hashimotos thyroiditis with coexistent papillary carcinoma and non-hodgkin lymphoma-thyroid. *Annals of Medical and Health Sciences Research*.

[14] Khalil M. O., Morton L. M., Devesa S. S. (2014). Incidence of marginal zone lymphoma in the United States, 2001–2009 with a focus on primary anatomic site. *British Journal of Haematology*.

[15] Fisher R. I., Dahlberg S., Nathwani B. N., Banks P. M., Miller T. P., Grogan T. M. (1995). A clinical analysis of two indolent lymphoma entities: Mantle cell lymphoma and marginal zone lymphoma (including the mucosa-associated lymphoid tissue and monocytoid B-cell subcategories): a Southwest oncology group study. *Blood*.

[16] Determann O., Hoster E., Ott G. (2008). Ki-67 predicts outcome in advanced-stage mantle cell lymphoma patients treated with anti-CD20 immunochemotherapy: results from randomized trials of the European MCL Network and the German Low Grade Lymphoma Study Group. *Blood*.

[17] Møller M. B., Pedersen N. T., Christensen B. E. (2006). Mantle cell lymphoma: Prognostic capacity of the Follicular Lymphoma International Prognostic Index. *British Journal of Haematology*.

[18] Hoster E., Dreyling M., Klapper W. (2008). A new prognostic index (MIPI) for patients with advanced-stage mantle cell lymphoma. *Blood*.

[19] Chen R., Sanchez J., Rosen S. T. (2016). Clinical management updates in mantle cell lymphoma. *Oncology*.

[20] Rosenbluth B. D., Yahalom J. (2006). Highly effective local control and palliation of mantle cell lymphoma with involved-field radiation therapy (IFRT). *International Journal of Radiation Oncology, Biology, Physics*.

[21] Martin P., Chadburn A., Christos P. (2009). Outcome of deferred initial therapy in mantle-cell lymphoma. *Journal of Clinical Oncology*.

[22] Ruan J., Martin P. (2016). Which patients with mantle cell lymphoma do not need aggressive therapy. *Current Heart Failure Reports*.

